# Detection of Heavy Metals in Water Using Graphene Oxide Quantum Dots: An Experimental and Theoretical Study

**DOI:** 10.3390/molecules26185519

**Published:** 2021-09-11

**Authors:** Lorenzo Gontrani, Olivia Pulci, Marilena Carbone, Roberto Pizzoferrato, Paolo Prosposito

**Affiliations:** 1 Dipartimento di Chimica, Università di Roma “La Sapienza”, P. le A. Moro 5, 00185 Roma, Italy; 2 Dipartimento di Fisica, Università di Roma “Tor Vergata”, Via Della Ricerca Scientifica, 00133 Roma, Italy; olivia.pulci@uniroma2.it; 3 Dipartimento di Scienze e Tecnologie Chimiche, Università di Roma “Tor Vergata”, Via Della Ricerca Scientifica, 00133 Roma, Italy; carbone@uniroma2.it; 4 Dipartimento di Ingegneria Industriale, Università di Roma “Tor Vergata”, Via del Politecnico 1, 00133 Roma, Italy; paolo.prosposito@uniroma2.it

**Keywords:** graphene oxide, quantum dots, TD-DFT, absorption, fluorescence, quenching, enhancement, concentration, optical response, heavy metals

## Abstract

In this work, we investigate by ab initio calculations and optical experiments the sensitivity of graphene quantum dots in their use as devices to measure the presence, and concentration, of heavy metals in water. We demonstrate that the quenching or enhancement in the optical response (absorption, emission) depends on the metallic ion considered. In particular, two cases of opposite behaviour are considered in detail: Cd${}^{2+}$, where we observe an increase in the emission optical response for increasing concentration, and Pb${}^{2+}$ whose emission spectra, vice versa, are quenched along the concentration rise. The experimental trends reported comply nicely with the different hydration patterns suggested by the models that are also capable of reproducing the minor quenching/enhancing effects observed in other ions. We envisage that quantum dots of graphene may be routinely used as cheap detectors to measure the degree of poisoning ions in water.

## 1. Introduction

Heavy metals (HMs) have become a considerable factor of contamination of drinking water due to growing global industrialization, diffuse agricultural exploitation of land, and, in some areas, natural occurrence with geological origin. Despite increasingly strict regulations and legislative actions, millions of people worldwide are exposed to high levels of HM intake via drinking water, while it is estimated that at least one-million suffer from chronic poisoning in developing countries [1]. Moreover, the presence of HMs in surface waters and seawater contributes to the risks for human health through food chain accumulation. The highest level of hazard comes from species like cadmium, lead, chromium, arsenic, and mercury, which are systemic toxicants producing multiple organ damage and are classified as human carcinogens even at very low concentrations [2]. For this reason, the Guidelines for Drinking-Water Quality by World Health Organization (WHO) [3] have set the threshold limits in the ppb range, e.g., 3 μg/L for Cd and 10 μg/L for Pb. Determination of such low levels of concentration is generally performed by using well-established laboratory methods including, but not limited to, atomic high-performance liquid chromatography (HPLC), absorption spectrophotometry (AAS), and inductively coupled plasma mass spectrometry (ICP-MS) [4,5]. However, these techniques require expensive and heavy instrumentations, well-trained staff, and complex time-consuming procedures. These factors obviously hamper a diffuse monitoring of water quality, especially in developing countries.

In recent years, it has been shown that nanomaterials possess remarkable optical, magnetic, and electrical properties that can be exploited in many technological fields, ranging from electronics [6,7] to biology and medicine [8,9]. Focusing on pollution-related issues, nanomaterial-based sensors have recently demonstrated to be promising candidates for the development of simple, environmentally friendly, and affordable methods for HM monitoring, as well as radiation-absorbing materials to be employed to fight electromagnetic pollution [10]. Based on colorimetric and fluorometric responses, optical sensing is compatible with water solutions and is suitable for applications in a variety of portable systems, ranging from cheap paper-based detection to cost-effective handheld smartphone-based devices [11]. Graphene-like carbon-based nanoparticles, generally referred to as carbon dots (CDs), have recently been investigated for their sensitivity to several analytes, including neutral molecules [12] and HM ions, coupled with high stability and very low toxicity [13,14]. Sensitivity of CDs has been studied by several groups by recording the fluorescence quenching that occurs in the presence of HM ions in water solution [15]. Multiple and cross-sensitivity, however, limited the selective detection and discrimination of different species. We have recently investigated the sensing properties of carbon nanoparticles synthesized by cage-opening and unfolding C60 fullerene via a modified Hummers reaction [16,17]. Sensitivity to HM ions in water was characterized through variations of both absorbance and fluorescence intensity, which enabled multiple and selective sensing of copper, lead, arsenic, and cadmium in the same water solution. In the present contribution, we attempt at rationalizing the difference in such sensitivity using Density Functional Theory (DFT) and Time-Dependent Density Functional Theory (TD-DFT) calculations of the structures of ground and excited states of the nanoparticles and of their absorption and fluorescence spectra, with particular regard to the different solvent effect experienced by cadmium and lead cations quantum dots water dispersions that show the most pronounced concentration effect in their emission intensities. The theoretical assessment of absorption spectra of toxic heavy metals (Cd, Hg, and Pb) and CDs was discussed in two papers by Shtepliuk et al. [18,19], whereas the fluorescence spectra of solutions of cations and carbon quantum dots derived from tea were very recently investigated theoretically by Hu et al. [20]. The latter study, though, focuses on lighter cations and does not consider the heavy metals Pb${}^{2+}$, and, most importantly, Cd${}^{2+}$, the ion that gives the large signal enhancement that we report in the present work. Moreover, some discrepancies between experiment and theory are highlighted in [20]. It is worth mentioning that the TEM structures there reported show the presence of various aggregates, whereas our systems have more uniform structures and contain only C, O, and H atoms. We therefore decided to (re-)examine the matter and, as far as we are aware, this is the first study that tries to assess the combined variations of both absorption and fluorescence spectra.

## 2. Materials and Methods

### 2.1. Experimental

**Figure 1:**
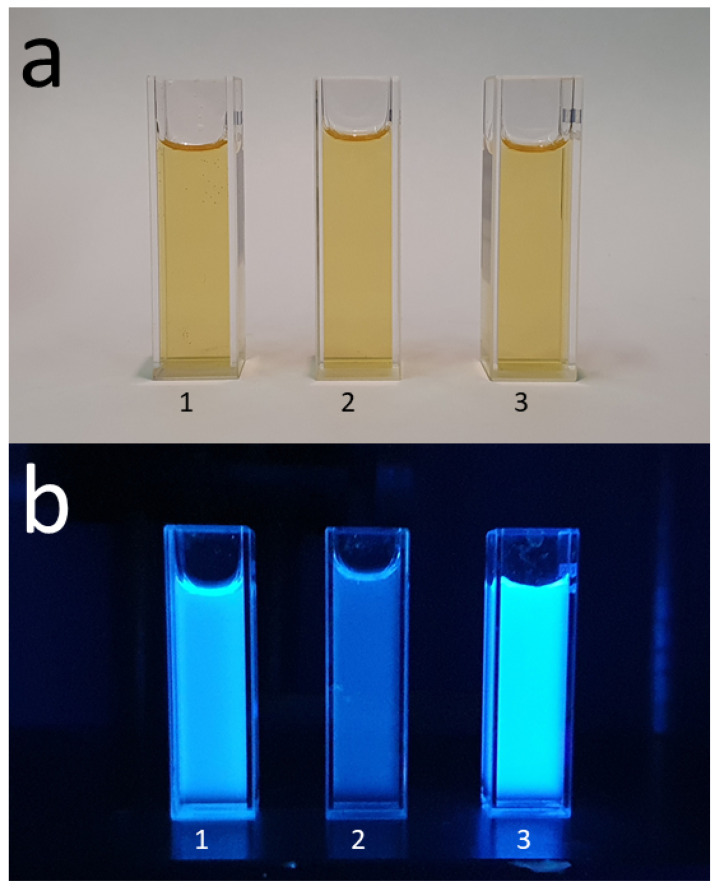
Photographs of UFQD sensing solution at a concentration of 50 μM: (1) pure and (2) after addition of Pb${}^{2+}$ or (3) Cd${}^{2+}$ at a concentration of 100 μM taken under visible light (**a**) and 365 nm UV light (**b**). The quantum yield (QY) of UFQD decreased to 45% after addition of Pb${}^{2+}$ and increased by 110 % after addition of Cd${}^{2+}$, approximately.

Synthesis of unfolded fullerene quantum dots (UFQDs) is described in more detail elsewhere [16,17]. Briefly, the Hummers method consists in a strong oxidation of fullerene performed by using highly-oxidant agents (NaNO_3_, KMnO_4_, and H_2_SO_4_) and a specific sequence of temperature variations that produce the opening of C_60_ cage. Subsequent purification through dialysis resulted in a stable water dispersion of monodisperse UFQDs nanoparticles (see Figure 1) with lateral dimensions in the range from 2 to 4 nm, as shown by dynamic light scattering (DLS) and TEM and SEM images. Extensive characterization by NMR, Atomic Force Microscopy (AFM), X-ray Photoelectron Spectroscopy (XPS), Fourier Transform Infrared Spectroscopy (FTIR), X-ray diffraction (XRD), and Raman spectroscopy confirmed that the nanoparticles had a main structure made of reduced graphene oxide (rGO) with the presence of a relatively low number of oxygen-containing functional groups [16,17]. The as-prepared water dispersion was diluted with deionized water to obtain a carbon nanoparticle concentration of 50 μM that is therein referred to as UFQD stock solution.

Experimental determination of the sensitivity of UFQDs was performed by mixing 1 mL of UFQD stock solution with 1 mL of the HM-salt solution at the appropriate concentration in deionized water. After gentle stirring for 30 s, absorbance and fluorescence spectra were recorded. UV–Vis absorption spectra were measured with a Cary 50 spectrophotometer (Varian Inc., Palo Alto, CA, USA) while fluorescence spectra were recorded with a laboratory setup for photoluminescence measurements [21]. The excitation wavelength $\lambda_{exc}$ = 300 nm was used for fluorescence spectra with a spectral passband of 2 nm for both excitation and emission light. The spectral response of the PL setup was calibrated over the visible wavelength range through a certified spectral fluorescence standard kit (Sigma-Aldrich, Saint Louis, MO, USA). The quantum yields (QY) of UFQD samples were estimated by using standard reference fluorophore solutions, i.e., both quinine sulfate and 9,10-diphenyl-anthracene (DPA) with an absorbance <0.05 OD (Optical Density) in the whole excitation/emission range. Extensive characterization by TEM, AFM, DLS, FTIR, NMR, and XPS was carried out and is reported in our previous studies [16,17].

### 2.2. Computational

We performed calculations on fragments of graphene, without and with metal ions, using DFT for geometry optimizations and control vibrational frequencies calculations. Time-Dependent DFT (TD-DFT) was instead employed to determine the electronic excited state energies and the absorption spectra. Optical emission spectra were obtained as absorption spectra using the optimized excited state geometries. The density functional ωB97X-D was employed, using the split-valence basis set 6-31+G(d) for light atoms [22] and the Los Alamos National Laboratory basis sets (including effective-core potentials), known as LANL2DZ [23], for Cd and Pb. The long-range corrected hybrid density functional chosen includes corrections for dispersion interactions and has proven effective in the calculation of geometries and excitation energies [24] All the calculations were performed with the Gaussian 16 program [25]. Post-processing analysis to obtain the electronic spectra was accomplished with the program Gaussview [26] using a Half-Width-Half-Height (HWHH) broadening of 0.333 eV for each transition, whereas MultiWfn [27] was used for further wavefunction analyses to gather information about the charge transfer in the transition (see Supplementary Material).

As experimental data were gathered on nanoparticles water suspensions, the solvent effect was taken into account with the Polarizable Continuum Model (PCM) [28], choosing water as solvent (static dielectric permittivity ϵ = 78.3553, permittivity at infinite frequency ϵ(∞) = 1.7778) and with explicit water molecules. More details on PCM method are given in the Supplementary Material. The validity of the optimization was checked through the calculations of vibrational frequencies, that reported no imaginary values in every case, thus confirming that all the stationary points found were real minima on the potential energy surface.

## 3. Results and Discussion

**Figure 2:**
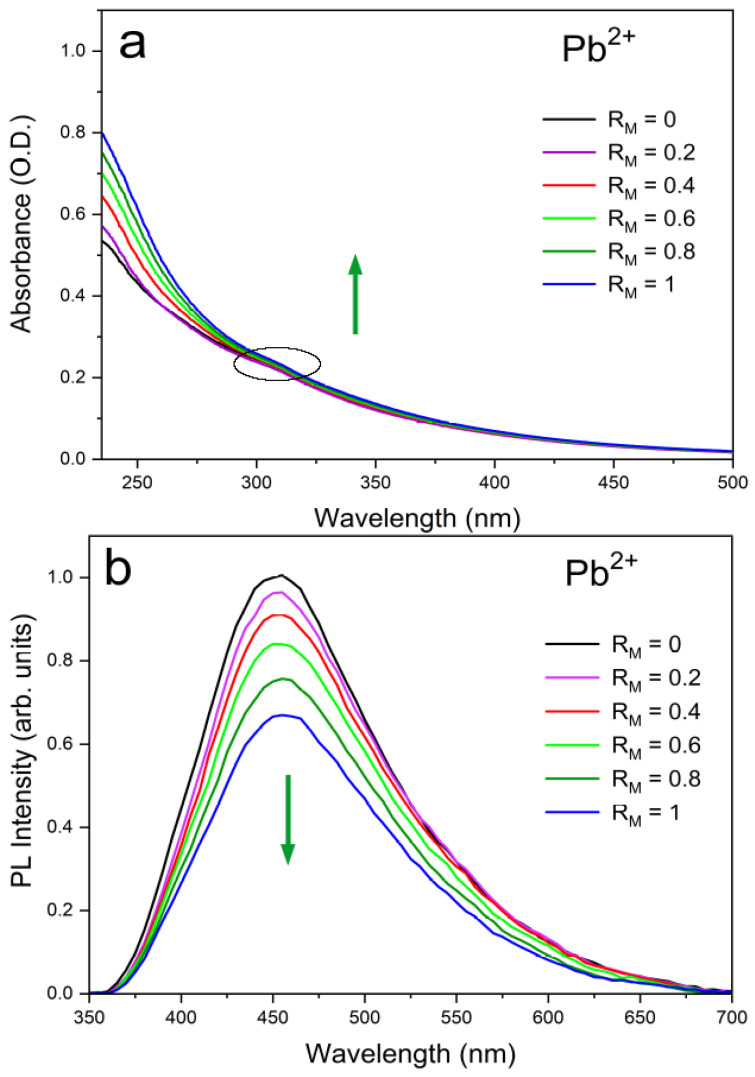
Experimental optical absorption (**a**) and emission (**b**) spectra of the aqueous suspension of UFQDs in the presence of Pb${}^{2+}$ at different values of the ratio of the respective molar concentrations RM = [Pb${}^{2+}$]/[UFQD]. A maximum decrease to 67% in the quantum yield was observed at RM = 1.

**Figure 3:**
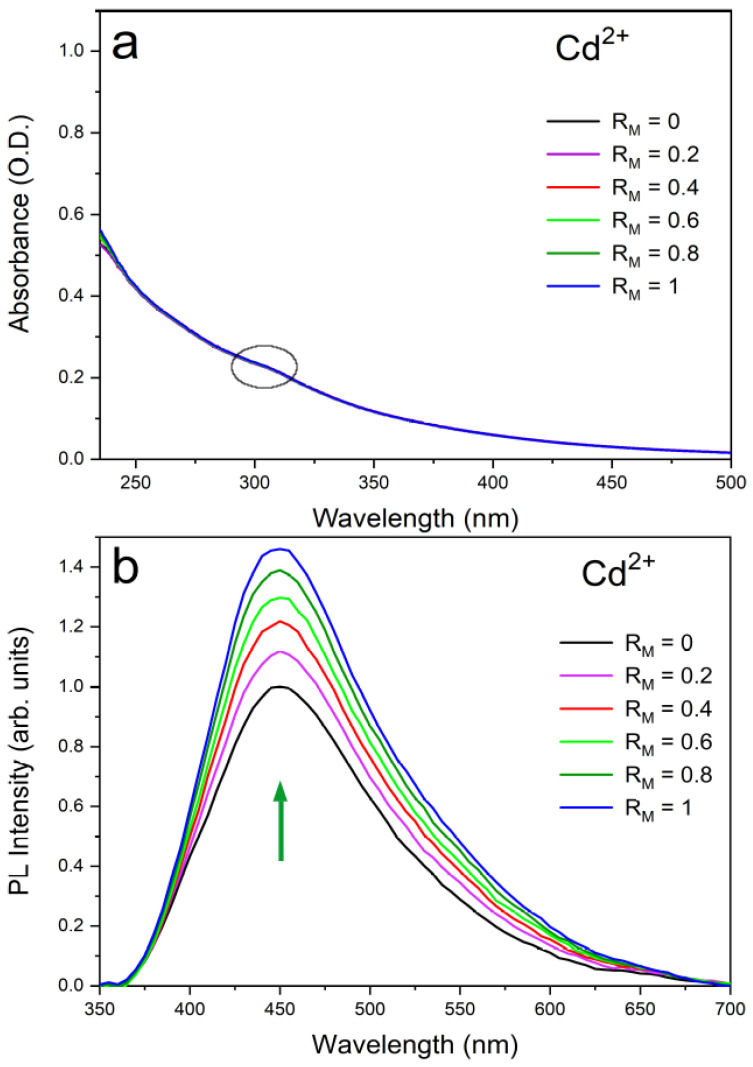
Experimental optical absorption (**a**) and emission (**b**) spectra of the aqueous suspension of UFQDs in the presence of Cd${}^{2+}$ at different values of the ratio of the respective molar concentrations RM = [Cd${}^{2+}$]/[UFQD]. A maximum increase of 47% in the quantum yield was observed at RM = 1.

The experimental absorption and emission spectra of water dispersions of UFQDs in the presence of Pb${}^{2+}$ and Cd${}^{2+}$ at different ion concentrations are reported in Figure 2 and Figure 3, respectively, and are labeled as a (absorption) and b (emission). The choice of these two cations was suggested by the remarkable intensity trend with concentration observed in their absorption and emission spectra that could be profitably exploited in purposely designed sensors. Namely, Cd and Pb give opposite trends in the emission spectra with increasing ion concentration in water (Cd: enhancement; Pb: quenching). See Figure 1 and [16,17].

For Pb${}^{2+}$, this corresponds to a Stern–Volmer quenching constant K${}_{SV}$ = 10${}^{4}$ M${}^{-1}$ and a limit of detection LOD = 1 μM. For Cd${}^{2+}$, a negative Stern–Volmer constant can be estimated in the low concentration limit K${}_{SV}$ = − 6.4 × 10${}^{3}$ M${}^{-1}$ and a limit of detection LOD = 500 nM. By contrast, the effect of concentration on absorption spectra is limited to an intensity increase with the concentration for Pb${}^{2+}$ cation solutions below 300 nm, while is almost absent in the other cation solutions. In each picture, the spectra measured for the water dispersions of “bare” (non coordinated to metal, black line) UFQDs and cation-coordinated systems at different metal to graphene molar ratios (R${}_{M}$, pink to blue lines) are piled up (the arrows in the figures are a guide for the eyes). Regarding the emission spectra, it can be noticed that all the dispersions containing the same cation share the same wavelength (λ) for the peak maximum, that is, 455 nm for Pb${}^{2+}$ and 450 nm for Cd${}^{2+}$, whereas the absorption spectra do not show peaks in the 250–500 nm λ range, and only a faint shoulder at 305 nm can be pointed out in both systems and is generally attributed the *n*−$\pi^{*}$ transition in C=O bonds of oxygen-containing functional groups (dashed-line circle in Figure 2 and Figure 3). The observed fluorescence red-shift is, therefore, about 150 nm for both systems.

**Figure 4:**
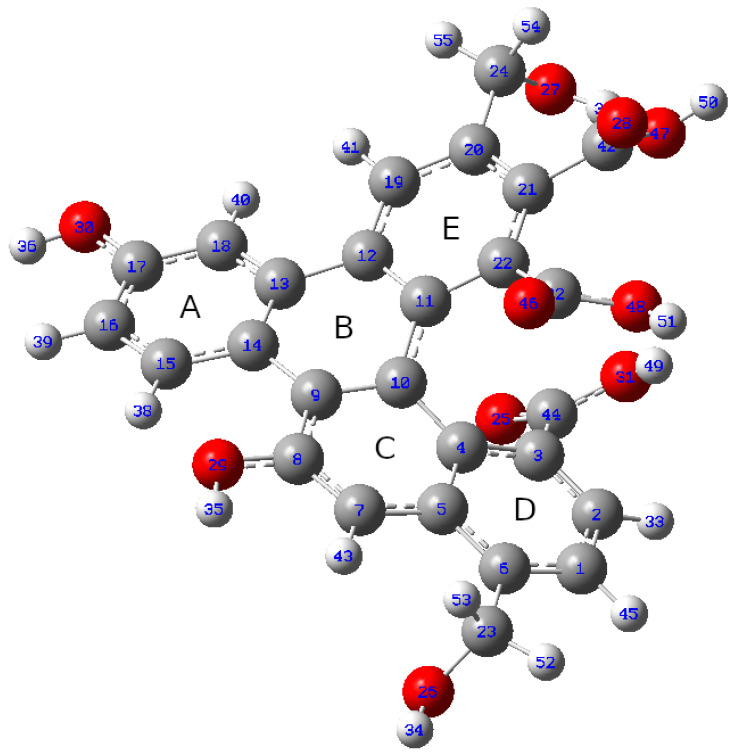
Optimized geometry of bare UFQD at ω-B97XD/6-31+G(d) level. Carbon: gray; hydrogen: white; oxygen: red.

The calculation of the absorption and emission spectra was accomplished according to the following protocol. The starting point consisted in a fragment of oxidized graphene that was obtained through fullerene unfolding (UFQD—see Section 2.1). The skeleton of this system is made up of five fused aromatic rings; identified by the letters A–E in Figure 4; three carboxyl groups (two on E-ring and one on D-ring, respectively); and four OH groups, two phenol-like fragments and two benzyl (CH_2_OH) ones, totaling 55 atoms. In the first step, the optimized structure of the oxidized graphene fragment in water solution (modeled by PCM) was obtained. The relative geometry is reported in Figure 4 and shows a sizable curvature if compared with the planar periodic arrangement of graphene sheets without substituents.

Such bending is observed both in vacuo and in the PCM model. Once the relaxed structure was obtained, a linear response TD-DFT calculation of the system was accomplished, thus obtaining the vertical transitions to 20 excited states both in vacuo and in PCM solution.

The UFQD fragment (host) was then further modified in order to allow for the interaction with a cation (guest). Given that the cations considered in this study are both divalent (charge +2), two carboxylic groups (one in ring E, one in ring D) were deprotonated to finally obtain a neutral system. Overall, three anchoring groups were available for binding near the cation: one COOH and two COO^−^. Cd and Pb cations were used to build the complex at first, whereas Ca${}^{2+}$ and As(III) were additionally included in a later phase in order to verify the validity of the model. These two ions were chosen as the first one shares with Cd${}^{2+}$ the non-common feature of intensity boost (with respect to the majority of the ions), but at a smaller extent, whereas As(III) another cation leading to fluorescence quenching like Pb${}^{2+}$ but possessing different oxidation state (+3) (See Ref. [17]). The most diffuse form of trivalent arsenic in water solution is the arsenite anion (AsO_3_${}^{3-}$); yet, for simplicity, we attempted at simulating the ion effect with the bare As${}^{3+}$ and with another non-protonated carboxylic group. These models, that will be labeled “UFQCD-Cd” (or Pb/Ca/As) hereafter, were treated in a similar way (geometry optimization and TD-DFT) as the non-complexed system, with the additional excited state relaxation after the absorption step. In this procedure, the potential energy surface (PES) of the first excited state was scanned in search of a local minimum and for every sampled point a TD-DFT calculation with 5 transitions was performed. Given the complexity of the calculation, characterized by very flat potential energy surfaces, the optimizer search step (in terms of change in geometrical coordinates) was greatly reduced from the default values up to 0.05 Bohrs and 0.05 radians. If the changes from step to step are not very drastic, the chances of swapping between the target excited state and other (wrong) excited states surfaces of similar energy is lower and the overall time needed for the optimization decreases, in spite of the smaller step adopted [29,30]. This way, the optimized structures could be obtained, satisfying the same convergence criteria as in the ground state. A larger TD-DFT calculation with 20 transitions was then run on the optimized excited state geometry. The calculated vertical transitions using this relaxed geometry correspond to the emission wavelengths and were calculated in PCM medium also for UFQD fragment not complexed with the cation for comparison. Finally, a further group of models was generated—UFQCD-Cd-WAT and UFQCD-Pb-WAT—where the solvent effect was taken into account explicitly, by adding three water molecules in the solvation shell of the ion. These models will be identified as “diluted models” as opposed to the “concentrated” ones. The choice of three water molecules was dictated by two main reasons: (a) most metal ions typically show six-fold coordination with water or other ligands, that would have been reached, at most, by the three graphene ligands and the three water molecules (b) the increase in atom count is sufficiently low to allow for the (very demanding) excited-state calculations.

The values of the highest wavelength in the calculated absorption and emission spectra are collected in Table 1. The calculated red-shifts (Stokes shifts) between absorption and emission fall in the 100–120 nm range and comply satisfactorily with experimental data, also considering the uncertainty in the position of the shoulder in the absorption spectra. Moreover, for every calculated transition, the coefficients (c) of the TDDFT expansion matrix containing one-electron promotions could be used to “assign” the transition, according to the following formula, valid for closed-shell configurations (like the systems considered):

(1) $$\%contrib=c^{2}*2*100$$

that yields the percent contribution of a given transition between ground state orbitals (e.g., HOMO→LUMO) to the excited state [31]. It should be noticed that the emission transitions have “purer” HOMO→LUMO character in any case, whereas the absorption lines of the ion-containing system are more contaminated by other transitions (mainly HOMO − 1→LUMO and HOMO→LUMO + 1), and the oscillator strength values of the transitions increase markedly. The increase of HOMO→LUMO character is probably brought about by the expunction of some states having energy degeneracy from the TDDFT interaction matrix upon the structure relaxation and/or by the relative transitions turning to forbidden.

**Figure 5:**
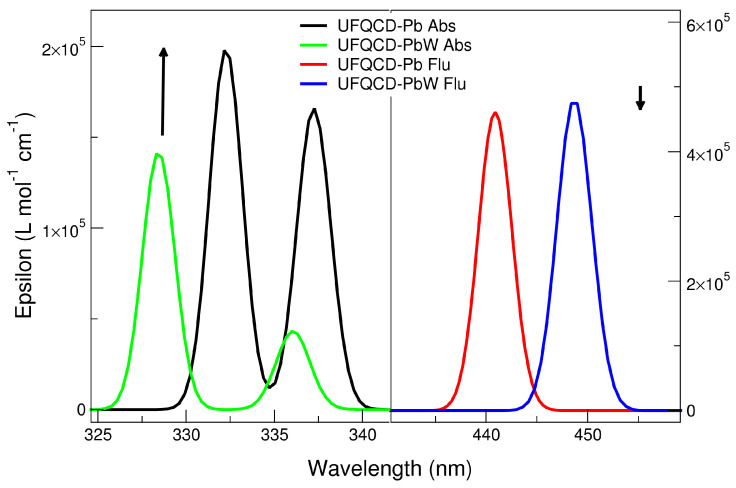
UFQD-Pb absorption (**left**) and emission (**right**) theoretical spectra.

**Figure 6:**
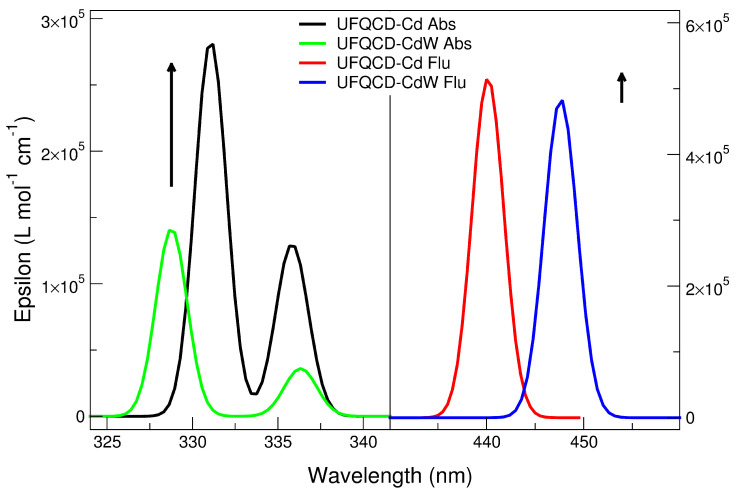
UFQD-Cd absorption (**left panel**) and UFQD-Cd emission (**right panel**) theoretical spectra.

**Figure 7:**
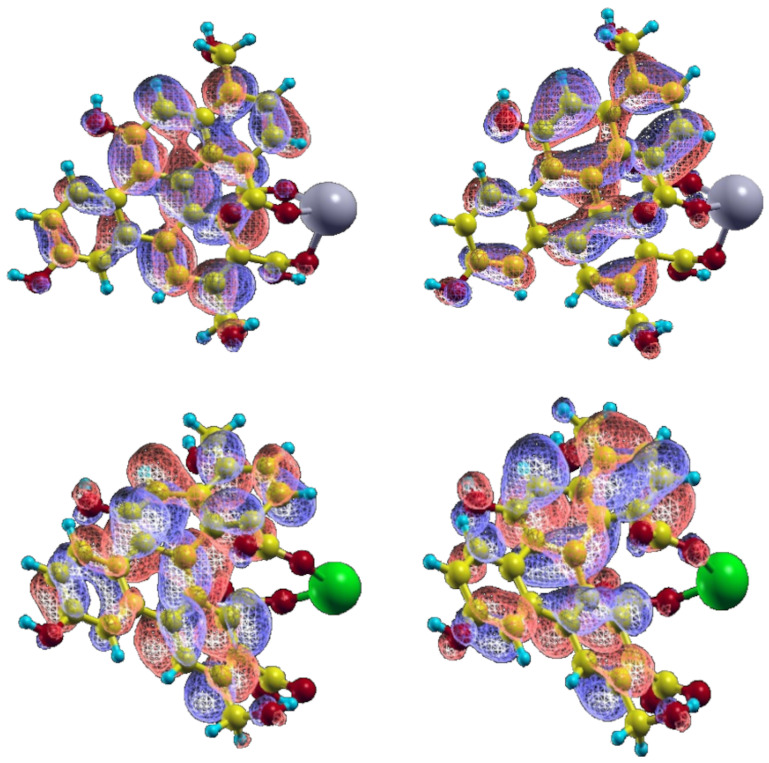
HOMO and LUMO density surfaces of UFQD-Pb (**top**) and UFQD-Cd (**bottom**). HOMO: right panel, LUMO: left panel.

**Figure 8:**
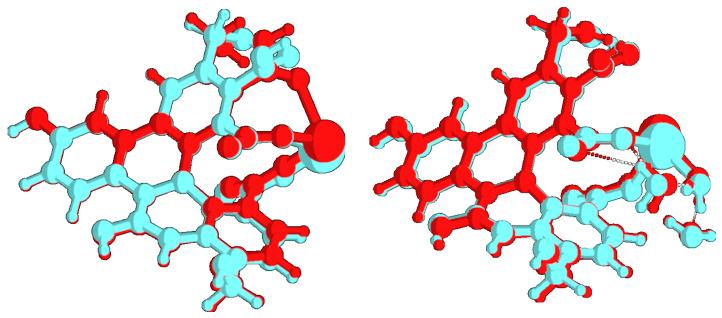
Overlay of UFQD-Cd/UFQD-Pb (**left panel**) and of UFQD-CdW/UFQD-PbW structures (**right panel**). Pb: red; Cd: cyan.

The data reported in Table 1 appear to be rather in agreement with most experimental trends. Actually, the absorption wavelength of the largest λ transition remains almost constant when passing from “diluted” to “concentrated” models in both ions’ spectra, and are not far from the wavelength values of the shoulder observed. Yet, the results are not straightforward, as the second-lowest transition is calculated to have the predominant HOMO-LUMO character in Cd${}^{2+}$ but not in Pb${}^{2+}$, so that a small shift (i.e., from 328 to 337 nm) would be predicted in this case. Overall, bearing in mind that the experimental pattern results from the convolution of bands of non-infinitesimal width, the analysis should not be focused on the single lines only, but should consider the entire peak(shape), and in this case, the agreement would be regarded as satisfactory (left panels of Figure 5 and Figure 6). The oscillator strength values, in turn, appear to rise somewhat for Pb${}^{2+}$ from 0.13 to 0.15/0.18 units, but this seems to happen for the other ion as well, opposite to the experimental finding. On the contrary, the emission data trends, where the concentration effect in the experimental patterns is much more evident, are reproduced quite correctly. Indeed, along the concentration increase, the opposite behaviour of the systems can be observed: fluorescence is quenched (Osc. Str. from 0.44 to 0.42) in presence of Pb${}^{2+}$ ions, while the signal is enhanced (from 0.45 to 0.47) with Cd${}^{2+}$. The extension of the analysis to Ca${}^{2+}$ and As(III) corroborated the validity of the model. In particular, in the first case, the calculation was successful (same behavior of Cd${}^{2+}$ but at a lower degree, as in [17]); in As(III), the experimental pattern was verified (quenching) but the obtained intensity decrease was quite low. This issue is probably related to the simplistic modeling of the structure with the As${}^{3+}$ cation instead of the (more computationally demanding) arsenite anion. An evident discrepancy with experimental data, though, was found in the calculation of the vertical absorption of the UFQD (PCM) solution without ions, that is wrongly predicted at 416 nm. The theoretical patterns obtained from the broadening of the calculated transition lines (see Section 2) are shown in Figure 5 (Pb${}^{2+}$) and Figure 6 (Cd${}^{2+}$), in the 320–350 nm range for absorption (left panel) and in the 430–460 range (right panel) for emission, covering this way the lowest energy part of both phenomena. In order to tentatively explain the observed trends, the HOMO and LUMO orbital densities of both absorption and emission ground states, as well as the total electron densities of ground and excited states and their differences were analyzed. The difference between LUMO and HOMO surfaces (reported in Figure 7), as well as the data reported in Table 1 suggest that the process can be classified as a $\pi \to \pi^{*}$ transition occurring within sp${}^{2}$-carbon domains of the graphene-like defective lattice. Interestingly, the HOMO and LUMO densities do not show large isovalues around the ion, but the latter is capable of inducing a different deformation of the conjugated π system nearby. In fact, Pb${}^{2+}$ establishes three interactions with negatively charged polar groups of the graphene nanoparticles, one with D ring COO^−^ group and two with E ring COOH and COO^−^ groups (Figure 7, top panel), whereas Cd${}^{2+}$ forms only two such interactions, as the E-ring COOH moiety interacts with the alcohol CH_2_OH group of ring E through a hydrogen bond (Figure 7, bottom panel). The latter interaction pattern results in a small uplifting (See Figure 8, left panel, cyan) of the A-B-E rings portion (the out-of-plane dihedral angle between B and E ring decreases about 5 degrees) that ultimately appears to favor the LUMO electron density transfer towards A and B rings (from the ion towards the left in Figure 7). This observation is confirmed by the variation of the total dipole moment calculated on the whole electron density of the excited states, that can be both largely described as ground state LUMOs (see Table 1), which increases by 1.22 Debye in Cd${}^{2+}$ and by 1.02 in Pb${}^{2+}$ with respect to the ground state. The calculation of the Mulliken partial charges differences between ground and excited states, as well as the calculation of the maps of Interfragment Charge Transfer (IFCT) [32] and of the Δr charge transfer descriptor by Guido et al. [33], shed more light on the matter (see Additional Material Section for details). In particular, the additional hydrogen bond interaction leads to a larger positive charge depletion in Pb${}^{2+}$ than in Cd${}^{2+}$ (1.13 vs. 1.51 electrons), mostly originated by the electron donation from E-ring COO^−^ group the lead cation is linked to. Overall, the increased coordination of the cation withdraws some negative charge from the graphene system, and the charge unbalancing occurring during the electronic transition is more limited to the polar groups interacting with the metal in Pb${}^{2+}$ system (the charge of E-ring COO^−^ carbon atom gets 0.03 units), whereas in Cd${}^{2+}$ QDOT a sizable charge variation is observed in most atoms of graphene rings. Such variation goes hand in hand with the (small) difference in the oscillator strengths calculated. When the UFQDs are “diluted”, as in the model with three explicit waters, the water cap surrounding the nanoparticle (from the ion side) prevents the COOH⋯CH_2_OH hydrogen bonding and the ring structures are largely superimposable, with the only small structural differences confined to the ion–water interactions. This geometrical arrangement is shown in Figure 8, where the structures of hydrated and non-hydrated models for both ions are superimposed. The main consequences of the structural coincidence of chromophores are that the two LUMO surfaces are substantially equivalent, the oscillator strengths are almost equal and the dipole moment variations are negligible (0.0014 and 0.058 Debye in Cd${}^{2+}$ and Pb${}^{2+}$, respectively).

## 4. Conclusions

In this paper, we propose a theoretical/computational explanation of the intensity features observed in the absorption and emission spectra of water dispersions of Unfolded Fullerene Quantum Dots in the presence of metal ions. The use of UFQDs within a spectrophotometric protocol for the detection of small concentrations of polluting heavy metal ions is a very effective and rather low-cost method of water analysis. The simulation protocol reported appears to be appropriate to reproduce, at least to a qualitative degree, the quenching effect of the fluorescence that occurs when Pb${}^{2+}$ concentration in the system increases, and the intensity rise experienced by Cd${}^{2+}$ dispersions with increasing cation content. The main reason leading to the opposite behaviors appears to reside in the different conformations of two rings of the graphene chromophore. In Cd${}^{2+}$ system, the cation is predicted to be less solvated (coordinated) by the carboxy polar groups than Pb${}^{2+}$ system, thus permitting the establishment of an intermolecular hydrogen bond with a alcohol group further apart, that pushes the surrounding carbon rings. This geometrical arrangement favors a larger HOMO–LUMO electron transfer and the increase of dipole moment increase and transition oscillator strength, in compliance with the higher fluorescence intensity. When the cation is surrounded by water molecules (dilution), the latter mechanism does not operate and the two systems are predicted to have a very similar behaviour.

## Figures and Tables

**Table 1:** Energy, wavelength, oscillator strengths, and percent contributions of HOMO-LUMO transition of the calculated absorption and emission spectra of graphene-ion systems. The letter W refers to model with explicit waters (diluted).

System	E (eV)	λ (nm)	Osc. Str.	% HOMO-LUMO
Absorption				
UFQD No Ion	2.98	416.23	0.1793	90.92
UFQD-Pb Concentrated	3.68	337.24	0.1526	55.00
${}^{\prime \prime }$	3.73	332.26	0.1829	27.51
UFQD-PbW Diluted	3.69	336.06	0.0397	5.26
${}^{\prime \prime }$	3.78	328.44	0.1307	79.39
UFQD-Cd Concentrated	3.69	335.83	0.1196	21.43
${}^{\prime \prime }$	3.75	331.07	0.2606	69.20
UFQD-CdW Diluted	3.69	336.33	0.0331	9.05
${}^{\prime \prime }$	3.77	328.77	0.1303	77.80
Emission				
UFQD No Ion	2.80	443.46	0.4264	94.89
UFQD-Pb Concentrated	2.81	440.89	0.4238	95.30
UFQD-PbW Diluted	2.76	448.70	0.4428	94.66
UFQD-Cd Concentrated	2.82	440.14	0.4743	95.24
UFQD-CdW Diluted	2.77	447.63	0.4457	94.78
UFQCD-As Concentrated	2.66	466.60	0.1906	92.59
UFQCD-AsW Diluted	2.58	479.79	0.1954	93.72
UFQCD-Ca Concentrated	2.80	443.05	0.4715	95.36
UFQCD-CaW Diluted	2.78	446.40	0.4559	95.32

## Data Availability

Not applicable.

## Supplementary Materials

The following Supplementary Material is available online, Details on experimental spectral response calculation. Figure S1: Atom numbering of the structure CQDOT-Cd${}^{2+}$+ with no explicit water molecules, Figure S2: Atom numbering of the structure CQDOT-Pb${}^{2+}$+ with no explicit water molecules, Figure S3: Atom numbering of the structure CQDOT-Cd${}^{2+}$+ with three explicit water molecules, Figure S4: Atom numbering of the structure CQDOT-Pb${}^{2+}$+ with three explicit water molecules. Table S1: Cartesian coordinates of the system CQDOT-Cd${}^{2+}$ with no explicit water molecules, Table S2: Cartesian coordinates of the system CQDOT-Pb${}^{2+}$ with no explicit water molecules, Table S3: Cartesian coordinates of the system CQDOT-Cd${}^{2+}$ with three explicit water molecules, Table S4: Cartesian coordinates of the system CQDOT-Pb${}^{2+}$ with three explicit water molecules. Details on PCM method and electronic spectra calculations; Wavefunction analysis: (1) Mulliken Partial charges of ground (GS) and excited (ES) states and their differences for models without explicit water; (2) Inter Fragment Charge Transfer (IFCT); (3) Details of Spectral Response calculation.

## Abbreviations

The following abbreviations are used in this manuscript:

DFT	Density Functional Theory
TDDFT	Time-Dependent Density Functional Theory
PCM	Polarizable Continuum Model
UFQD	Unfolded Fullerene Quantum Dots
HOMO	Highest Occupied Molecular Orbital
LUMO	Lowest Unoccupied Molecular Orbital
