# Supplementary material for: Detection of Heavy Metals in Water Using Graphene Oxide Quantum Dots: An Experimental and Theoretical Study
Source: Molecules. 2021 Sep 11;26(18):5519. doi: 10.3390/molecules26185519 (PMC8467530; doi:10.3390/molecules26185519)
Supplement: Supplementary file 1 [file molecules-26-05519-s001.zip › molecules-1372258-supplementary.pdf]

Supporting Information

Detection of heavy metals in water using graphene oxide quantum dots: an experimental and theoretical study

Lorenzo Gontrani*, Olivia Pulci, Marilena Carbone, Roberto Pizzoferrato* and Paolo Prosposito

Spectral Response calculation (experimental)

The spectral response of the PL setup was calibrated over the visible wavelength range trough a certified spectral fluorescence standard kit (Sigma-Aldrich). The quantum yields (QY) of UFQD samples were estimated by using standard reference fluorophore solutions, i.e. both quinine sulfate and 9,10-diphenyl-anthracene (DPA) with an absorbance < 0.05 OD in the whole excitation/emission range, and the well-established formula:

$$QY_{Sample} = QY_{Ref} \cdot \frac{I_{Sample}}{Abs_{Sample}} \cdot \frac{Abs_{Ref}}{I_{Ref}} \cdot \frac{n^2{}_{Water}}{n^2{}_{Solvent}}$$

where QY_{Ref} and I_{Ref} are the quantum yield and the integrated PL emission of the standard reference solution, n_{Solvent} the refractive index of the solvent and Abs_{Ref} the absorbance at the excitation wavelength of the standard reference solution, while QY_{Sample} and I_{Sample} are the quantum yield and the integrated PL emission of the UFQD solution, n_{Water} is the refractive index of water and Abs_{Sample} the absorbance at the excitation wavelength of the UFQD solution.

Figure S1 – Atom numbering of the structure CQDOT-Cd^{2+} with no explicit water molecules

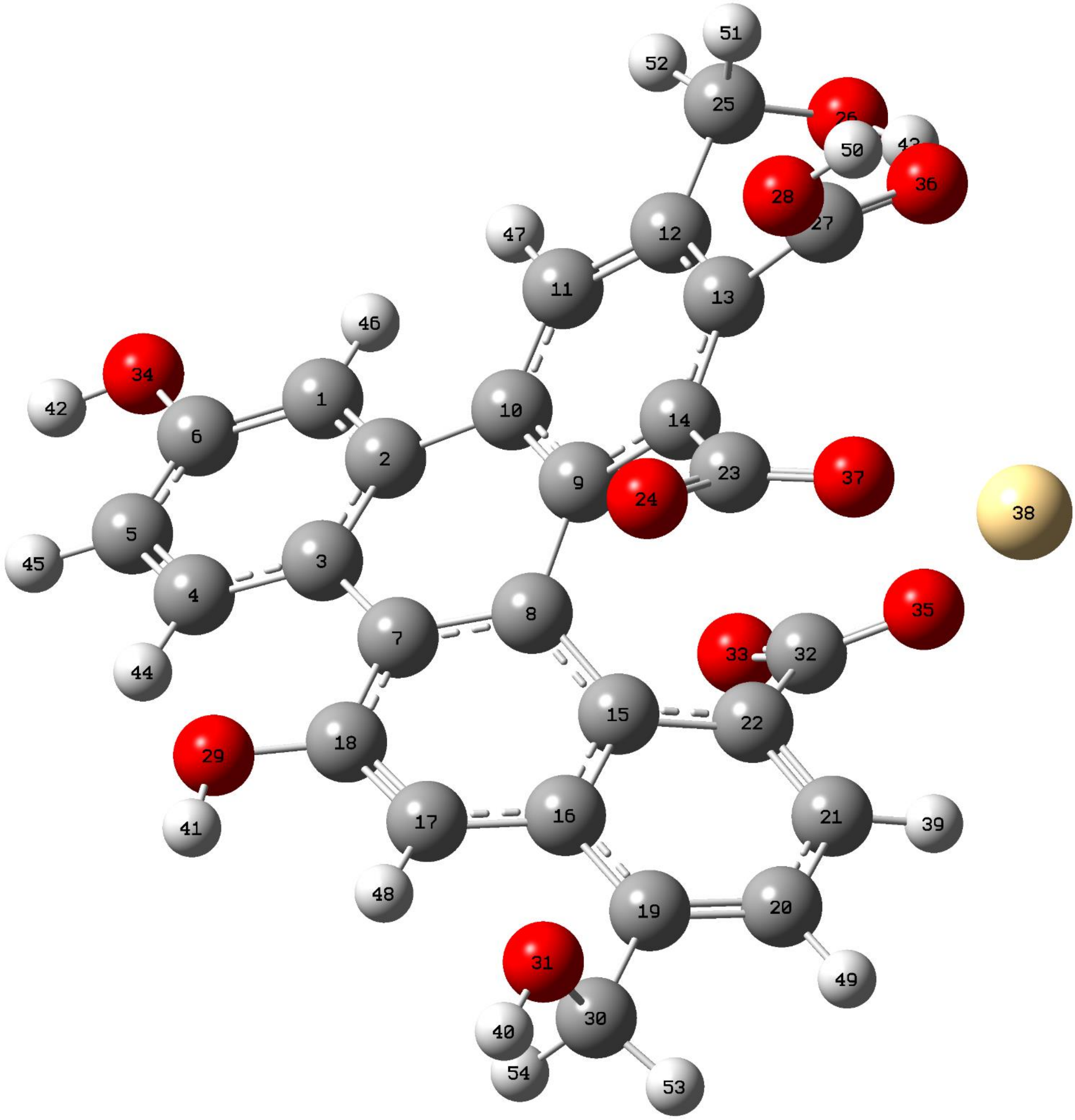

Tab S1 - Cartesian coordinates of the system CQDOT- Cd^{2+} with no explicit water molecules

Standard orientation:

Center Number	Atomic Number	Coordinates (Angstroms) X	Y	Z
C1	6	4.175966	-2.239372	-0.316437
C2	6	3.214433	-1.245096	-0.097400
C3	6	3.618494	0.115129	-0.087579
C4	6	4.975001	0.410968	-0.304559
C5	6	5.919395	-0.584749	-0.515562
C6	6	5.514287	-1.919038	-0.518565
C7	6	2.602229	1.151068	0.078933
C8	6	1.203967	0.775952	-0.023130
C9	6	0.834147	-0.539896	0.313848
C10	6	1.807840	-1.589268	0.176987
C11	6	1.373255	-2.911262	0.252810
C12	6	0.062697	-3.260212	0.565631
C13	6	-0.830172	-2.223331	0.926038
C14	6	-0.471550	-0.889799	0.821634
C15	6	0.243772	1.806924	-0.427992
C16	6	0.485157	3.134014	0.056973
C17	6	1.752226	3.395857	0.605018
C18	6	2.819359	2.458990	0.493247
C19	6	-0.502438	4.142773	-0.131353
C20	6	-1.620147	3.848850	-0.914248
C21	6	-1.764807	2.607842	-1.516473
C22	6	-0.819256	1.577130	-1.297382
C23	6	-1.372934	0.156268	1.460332
O24	8	-0.863070	0.933520	2.280089
C25	6	-0.360846	-4.710239	0.565609
O26	8	-1.217116	-5.029682	-0.528770
C27	6	-2.146037	-2.640833	1.507268
O28	8	-2.279464	-2.325195	2.793403
O29	8	4.070859	2.862234	0.822346
C30	6	-0.330339	5.520605	0.443199
O31	8	-0.285831	5.440781	1.867736
C32	6	-0.969400	0.306119	-2.117723
O33	8	0.014510	-0.107569	-2.749223
C34	8	6.377652	-2.954507	-0.714230
C35	8	-2.138472	-0.217578	-2.181007
C36	8	-2.985152	-3.284123	0.897737
C37	8	-2.620394	0.110208	1.183828
Cd38	48	-3.779919	-0.229165	-0.692570
H39	1	-2.619859	2.411142	-2.156693
H40	1	-0.103027	6.323828	2.214952
H41	1	4.061793	3.782293	1.120837
H42	1	7.277618	-2.623044	-0.836374
H43	1	-2.044177	-4.539512	-0.386200
H44	1	5.299824	1.442659	-0.321845
H45	1	6.960351	-0.320247	-0.682594
H46	1	3.914384	-3.290879	-0.326198
H47	1	2.069533	-3.722385	0.070328
H48	1	1.975979	4.384587	0.995522
H49	1	-2.375424	4.613712	-1.075567
H50	1	-3.154877	-2.621253	3.098462
H51	1	-0.850343	-4.979681	1.512420
H52	1	0.518481	-5.348874	0.457959
H53	1	-1.165793	6.154370	0.122667
H54	1	0.597667	5.976119	0.065698

Figure S2 – Atom numbering of the structure CQDOT-Pb[illegible] with no explicit water molecules

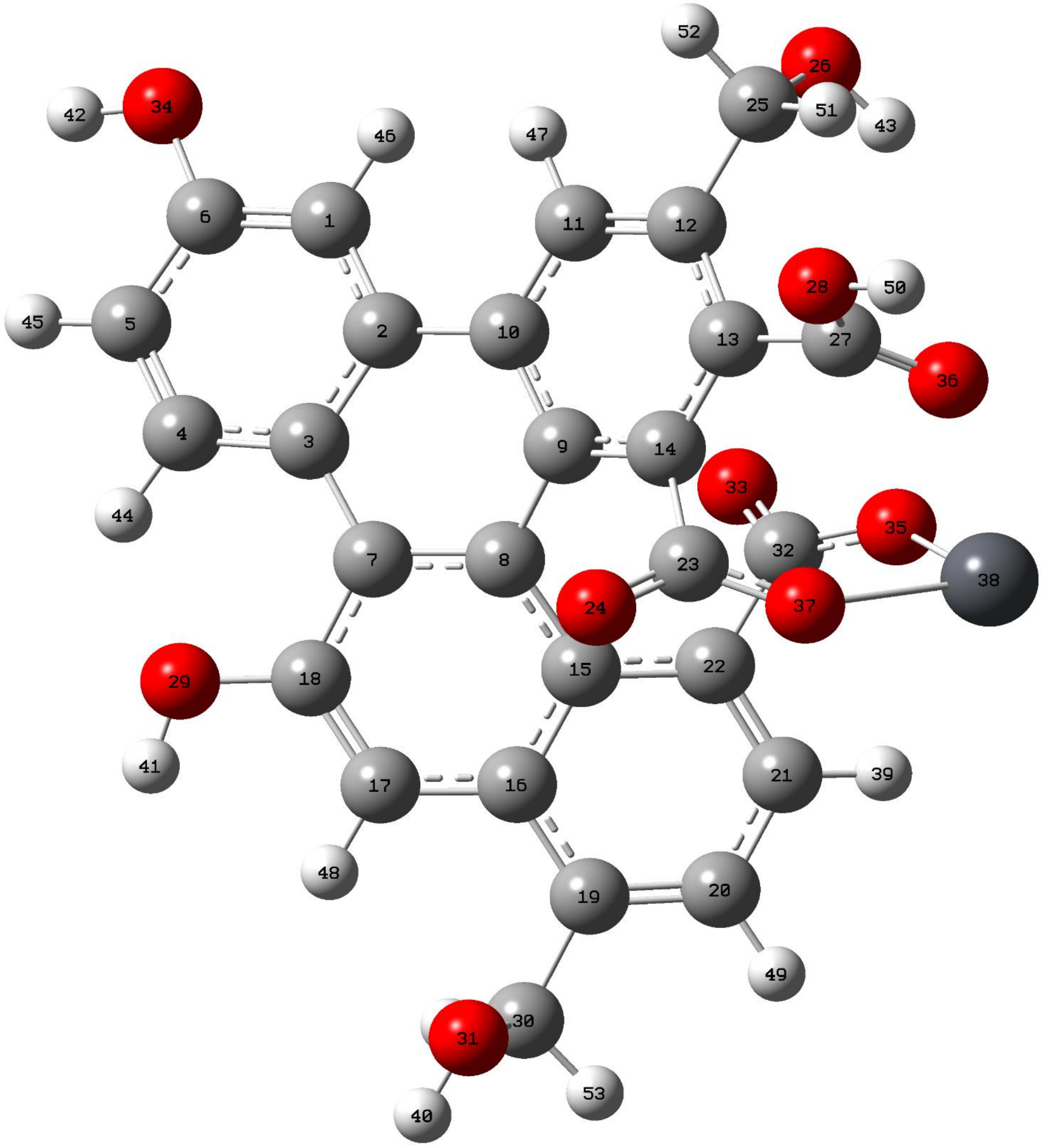

Tab S2 - Cartesian coordinates of the system CQDOT- Pb^{2+} with no explicit water molecules

Standard orientation:

Center Number	Atomic Number	Coordinates (Angstroms) X	Y	Z
C1	6	4.490609	-2.372840	-0.207198
C2	6	3.575222	-1.329552	-0.030633
C3	6	4.031432	0.012955	-0.096488
C4	6	5.397565	0.239135	-0.346222
C5	6	6.294731	-0.804136	-0.520875
C6	6	5.836946	-2.119944	-0.448185
C7	6	3.063267	1.096592	0.025675
C8	6	1.651135	0.769950	-0.015597
C9	6	1.233691	-0.511342	0.374534
C10	6	2.156404	-1.609085	0.259699
C11	6	1.656826	-2.899652	0.353451
C12	6	0.311424	-3.180236	0.622705
C13	6	-0.544881	-2.103152	0.916374
C14	6	-0.100781	-0.786091	0.857081
C15	6	0.706233	1.816973	-0.418654
C16	6	1.015992	3.154001	-0.020167
C17	6	2.320259	3.400726	0.446986
C18	6	3.343583	2.422000	0.350717
C19	6	0.047964	4.181943	-0.197222
C20	6	-1.131431	3.885535	-0.879921
C21	6	-1.357369	2.619176	-1.401415
C22	6	-0.429556	1.575647	-1.188594
C23	6	-0.897338	0.311509	1.522661
O24	8	-0.335511	1.070321	2.308872
C25	6	-0.156546	-4.613542	0.545283
O26	8	-0.229676	-5.078724	-0.802190
C27	6	-1.944872	-2.425864	1.331558
O28	8	-2.043506	-2.912131	2.548984
O29	8	4.619200	2.790146	0.612568
C30	6	0.303221	5.583774	0.284294
O31	8	0.478491	5.571677	1.699141
C32	6	-0.677631	0.262541	-1.901187
O33	8	0.199626	-0.232664	-2.602349
O34	8	6.651583	-3.197627	-0.606351
O35	8	-1.873115	-0.243338	-1.794071
O36	8	-2.930233	-2.305903	0.600267
O37	8	-2.177920	0.364464	1.289756
Pb38	82	-3.503806	-0.157093	-0.358487
H39	1	-2.246051	2.436060	-1.998899
H40	1	0.704818	6.466729	1.984273
H41	1	4.661114	3.728384	0.844618
H42	1	7.560537	-2.912632	-0.772300
H43	1	-0.868544	-4.533189	-1.281923
H44	1	5.765337	1.253160	-0.422976
H45	1	7.341994	-0.591716	-0.719639
H46	1	4.185558	-3.411293	-0.157276
H47	1	2.308648	-3.751916	0.196541
H48	1	2.591913	4.398416	0.779240
H49	1	-1.871178	4.666081	-1.034160
H50	1	-2.975665	-3.122452	2.745383
H51	1	-1.120614	-4.759106	1.045432
H52	1	0.567753	-5.269913	1.034099
H53	1	-0.543549	6.222821	0.007952
H54	1	1.201943	5.990263	-0.203403

Figure S3 – Atom numbering of the structure CQDOT-Cd2+ with three explicit water molecules

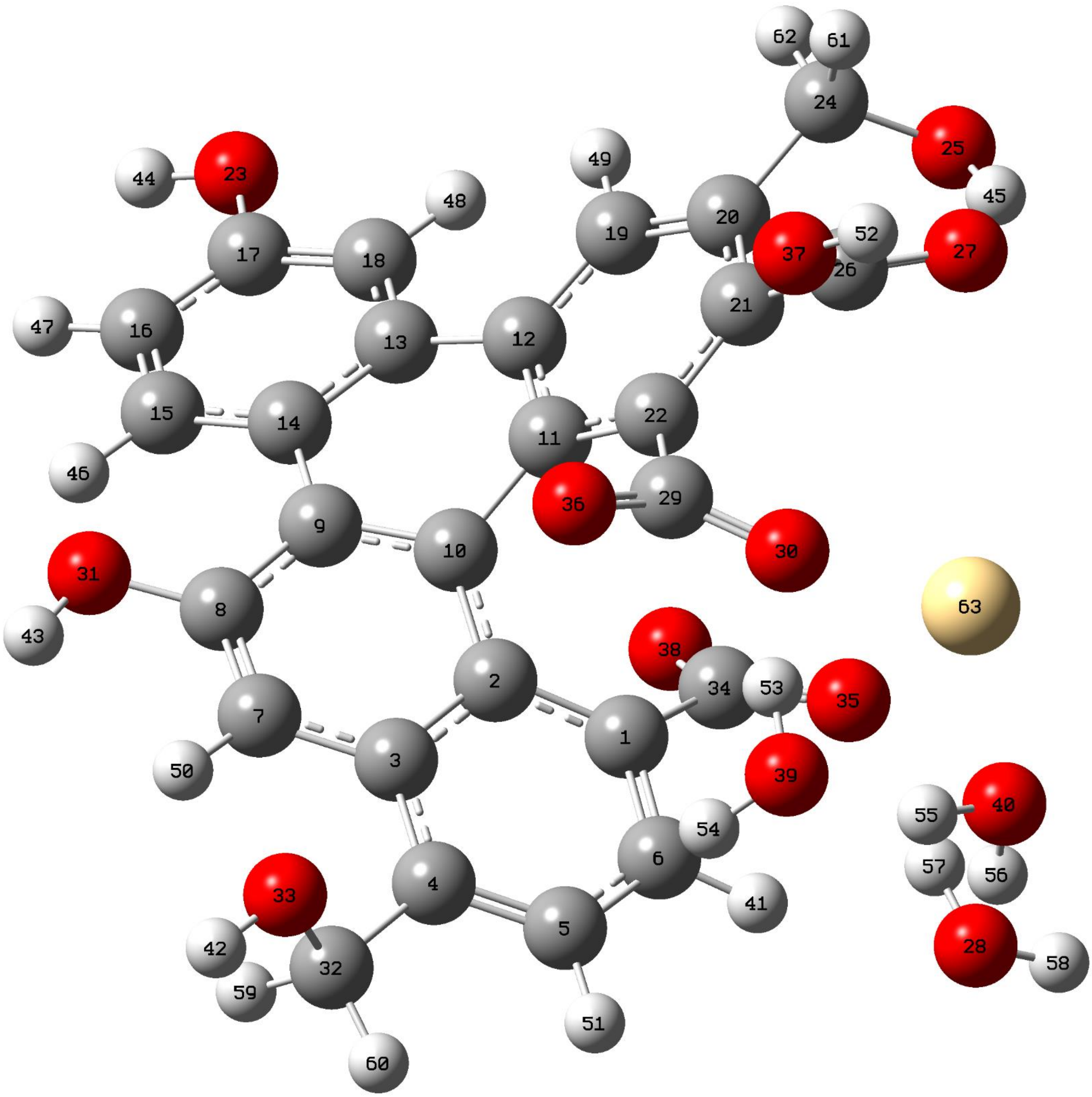

Tab S3 - Cartesian coordinates of the system CQDOT- Cd^{2+} with three explicit water molecules

Center Number	Atomic Number	Coordinates (Angstroms) X	Y	Z
C1	6	0.743012	1.470709	1.109898
C2	6	-0.310399	1.813539	0.265672
C3	6	-0.372847	3.133919	-0.291935
C4	6	0.770013	3.980519	-0.220286
C5	6	1.869177	3.554867	0.527845
C6	6	1.853142	2.342906	1.203417
C7	6	-1.619054	3.561157	-0.777317
C8	6	-2.807210	2.807186	-0.537791
C9	6	-2.757209	1.499908	-0.074603
C10	6	-1.428115	0.922219	-0.047452
C11	6	-1.285276	-0.441633	-0.386843
C12	6	-2.410241	-1.316877	-0.201105
C13	6	-3.698691	-0.762484	0.252112
C14	6	-3.895178	0.640874	0.236924
C15	6	-5.152130	1.147979	0.605014
C16	6	-6.192775	0.312290	0.986647
C17	6	-5.985920	-1.067204	1.017074
C18	6	-4.750300	-1.594039	0.657210
C19	6	-2.247605	-2.682223	-0.428178
C20	6	-1.058932	-3.237586	-0.888790
C21	6	0.001466	-2.358802	-1.207738
C22	6	-0.090286	-0.994527	-0.970827
O23	8	-6.952633	-1.950108	1.394344
C24	6	-0.946157	-4.733069	-1.079237
O25	8	-0.127216	-5.351059	-0.090902
C26	6	1.192370	-2.947886	-1.899197
O27	8	1.869775	-3.852833	-1.439421
O28	8	4.072837	0.878196	3.325617
C29	6	1.002945	-0.098059	-1.520891
O30	8	2.207447	-0.349220	-1.147513
O31	8	-4.004298	3.384460	-0.791643
C32	6	0.781263	5.333030	-0.873673
O33	8	0.618532	5.180702	-2.283283
C34	6	0.689452	0.272112	2.038903
O35	8	1.804113	-0.343612	2.262003
O36	8	0.697963	0.779224	-2.334669
O37	8	1.408815	-2.434923	-3.107594
O38	8	-0.375786	-0.005975	2.599222
O39	8	3.972439	1.703728	-1.408141
O40	8	4.833549	0.265864	0.744419
H41	1	2.702181	2.062713	1.815748
H42	1	0.549026	6.060168	-2.677422
H43	1	-3.882064	4.283169	-1.128471
H44	1	-7.765287	-1.479361	1.624115
H45	1	0.777495	-5.031572	-0.246276
H46	1	-5.315470	2.217998	0.611002
H47	1	-7.152510	0.734220	1.273401
H48	1	-4.636396	-2.670722	0.708420
H49	1	-3.079745	-3.358290	-0.264784
H50	1	-1.719876	4.552415	-1.210315
H51	1	2.740434	4.200820	0.611164
H52	1	2.204383	-2.848716	-3.485604
H53	1	3.261229	1.021612	-1.481417
H54	1	3.503937	2.518461	-1.178740
H55	1	4.629723	0.952479	0.064797
H56	1	4.752038	0.672730	1.635037
H57	1	3.227241	0.400546	3.189381
H58	1	4.552108	0.392375	4.009034
H59	1	-0.030943	5.955938	-0.468624
H60	1	1.729422	5.836702	-0.650501
H61	1	-0.569063	-4.974682	-2.082968
H62	1	-1.934283	-5.187651	-0.981785
Cd63	48	3.122605	-1.310414	0.699296

Figure S4 – Atom numbering of the structure CQDOT-Pb2+ with three explicit water molecules

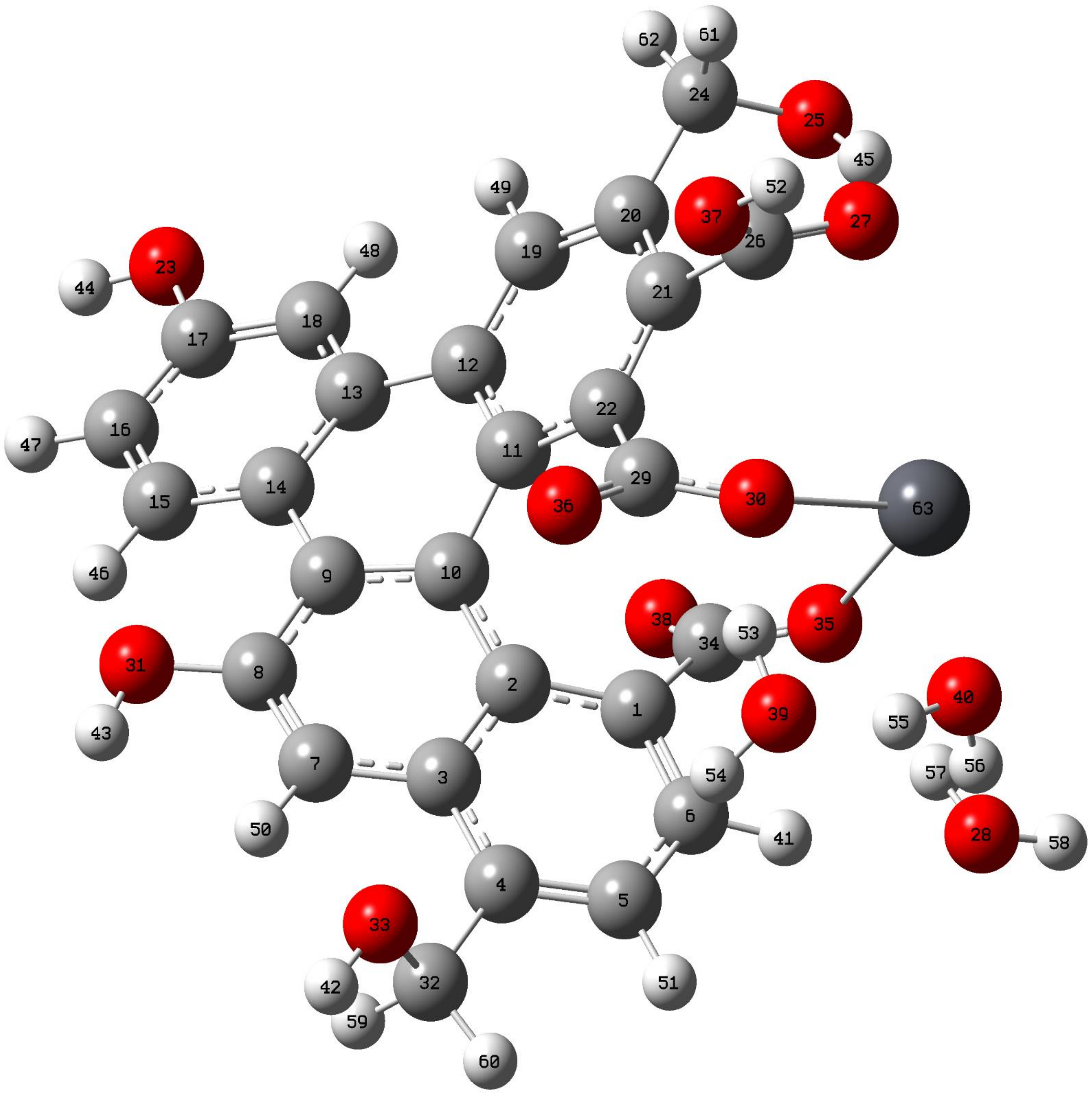

Tab S4 - Cartesian coordinates of the system CQDOT- Pb^{2+} with three explicit water molecules

Standard orientation:

Center Number	Atomic Number	Coordinates (Angstroms) X	Y	Z
C1	6	0.182720	1.721045	1.030300
C2	6	-0.957640	1.878997	0.248027
C3	6	-1.290260	3.175533	-0.265044
C4	6	-0.322581	4.218688	-0.212589
C5	6	0.872175	3.990000	0.473241
C6	6	1.117153	2.781083	1.107825
C7	6	-2.612502	3.368335	-0.694444
C8	6	-3.624240	2.392976	-0.440732
C9	6	-3.306920	1.105445	-0.027617
C10	6	-1.892362	0.795834	-0.057826
C11	6	-1.498267	-0.504749	-0.441904
C12	6	-2.430776	-1.586323	-0.266540
C13	6	-3.781822	-1.303691	0.249073
C14	6	-4.246179	0.034319	0.287304
C15	6	-5.562304	0.276357	0.714681
C16	6	-6.402044	-0.755725	1.109485
C17	6	-5.927788	-2.068270	1.094945
C18	6	-4.630966	-2.334452	0.670472
C19	6	-2.025047	-2.887982	-0.554755
C20	6	-0.767229	-3.195360	-1.062668
C21	6	0.100211	-2.123200	-1.354448
C22	6	-0.228619	-0.809659	-1.047832
O23	8	-6.682873	-3.130885	1.488526
C24	6	-0.360965	-4.630794	-1.303530
O25	8	0.556758	-5.105698	-0.322583
C26	6	1.372065	-2.443379	-2.078002
O27	8	2.263097	-3.129686	-1.600284
O28	8	3.599839	1.696291	3.204053
C29	6	0.717140	0.266253	-1.529523
O30	8	1.960227	0.153171	-1.144617
O31	8	-4.916412	2.733537	-0.634725
C32	6	-0.594894	5.561408	-0.829075
O33	8	-0.811681	5.401350	-2.229799
C34	6	0.402754	0.500407	1.888688
O35	8	1.643318	0.087748	1.998933
O36	8	0.334165	1.148239	-2.289422
O37	8	1.425151	-1.937381	-3.302182
O38	8	-0.529069	-0.008813	2.501867
O39	8	3.417049	2.490103	-1.409430
O40	8	4.297218	1.025850	0.669825
H41	1	2.032091	2.647631	1.673841
H42	1	-1.067891	6.257439	-2.597363
H43	1	-4.986374	3.651175	-0.933909
H44	1	-7.554720	-2.833534	1.781854
H45	1	1.384706	-4.612809	-0.442548
H46	1	-5.931836	1.292650	0.759488
H47	1	-7.413068	-0.536601	1.443266
H48	1	-4.305080	-3.367918	0.689568
H49	1	-2.712389	-3.712638	-0.401887
H50	1	-2.916176	4.330103	-1.097874
H51	1	1.609995	4.786130	0.539254
H52	1	2.283304	-2.162098	-3.704466
H53	1	2.762356	1.775091	-1.561900
H54	1	2.889495	3.268460	-1.182314
H55	1	4.058102	1.711189	-0.010335
H56	1	4.198512	1.427008	1.569211
H57	1	2.840423	1.083307	3.181092
H58	1	4.172020	1.397351	3.923062
H59	1	-1.481237	6.017237	-0.361867
H60	1	0.257398	6.226439	-0.646348
H61	1	0.063539	-4.758179	-2.309122
H62	1	-1.238485	-5.276635	-1.233884
Pb63	82	2.985319	-0.909024	0.551669

Details on PCM method and electronic spectra calculations

PCM – Polarizable Continuum Model (of solvation)

According to the model, the charge distribution of the target molecule (or group of molecules/ions) identified as solute, is embedded into a given volume of space (the ``molecular cavity'') dug into an infinite continuum dielectric (the solvent) characterized by specific macroscopic properties (density, refractive index, dielectric permittivity ε, etc.). The field produced by the solute charges into the exterior volume interacts with the solvent itself (described as a polarizable dielectric), and leads to the establishment of an apparent surface charge distribution ζ on the cavity surface. A new field, called reaction field, is produced in response to such charge and it can ultimately modify the previous solute charge distribution. The combination of solute and solvent reaction fields can be analytically treated, so that a final state in which both distributions are mutually equilibrated is obtained.

Within the quantum mechanical framework, the interactions between solvent and solute are modeled through specific (perturbation or reaction) operators of the solute Hamiltonian.

The Schrödinger equation describing the system becomes

$$(H^0 + V^{Reac})|\psi >= E|\psi >$$

where H^0 is the Hamiltonian in the absence of the solvent.

The solvent operator V^{Reac} acting on ψ depends on the surface apparent charge ζ and ultimately, as said before, on the solute charge distribution (i.e. the solute wavefunction ψ). This mutual interactions between ψ and V^{Reac} assures that the solution of the equation represents an equilibrated solute–solvent system.

Calculation of electronic spectra

Regarding the electronic transitions, the Gaussian program outputs the energy/wavelength value of the transition as well as the ``Oscillator Strength'' f, an adimensional quantity that is related to the Dipole Strength D stored internally by the relation

$$f_i = \frac{8\pi^2 \overline{\nu_i} m_e c}{3he^2} D_i$$

In turn, the dipole strength is linked to $\bar{\nu}$, the excitation energy (in wavenumbers, i. e. the reciprocal of wavelength) and to ϵ_i^{max}, the intensity at the maximum (i. e. when the incident radiation $\bar{\nu} = \bar{\nu_i}$) by

$$D_i = 4 \cdot \left[\frac{3 \cdot 1000 \cdot ln(10) \cdot hc}{32\pi^3 N}\right] \cdot \epsilon_i^{max}\sqrt{\pi}\frac{\sigma}{\bar{\nu_i}}$$

In these equations N is the Avogadro constant, e is the electron charge, m_e the electron mass, c the speed of light, h the Planck constant and σ is the standard deviation (bandwidth) equal to the half-width of the band when $\epsilon = \epsilon^{max}/e$ if the band shape is Gaussian.

To obtain simulated UV-Vis spectra comparable to experimental ones, the software Gaussview was used. The code assigns a Gaussian band shape

$$\epsilon_i(\bar{\nu}) = \epsilon_i^{max} exp\left[-\left(\frac{\bar{\nu} - \bar{\nu_i}}{\sigma}\right)\right]^2$$

to every calculated absorption/emission transition and plots the convolution of all the bands. For the calculations of these spectra, the broadening assigned to each transition was 0.333 eV (as Half Width at Half Height).

See the webpage https://gaussian.com/uvvisplot/ for further details-

Wavefunction analysis

1) Mulliken Partial charges of ground (GS) and excited (ES) states and their differences for models without explicit water.

The charge of hydrogen atoms is condensed in the heavy atom. Refer to figures/tables S1 and S2 for atom labeling.

	Cd GS	Cd ES	Delta	Pb GS	Pb ES	Delta	Type
C1	-0.913075	-0.892919	0.020156	-0.988626	-0.964650	0.023976	Aring
C2	0.468504	0.452193	-0.016311	0.468772	0.450918	-0.017854	Bring
C3	0.484823	0.454373	-0.03045	0.474850	0.449949	-0.024901	Bring
C4	-0.147725	-0.139927	0.007798	-0.109829	-0.101197	0.008632	Aring
C5	0.036759	0.024034	-0.012725	-0.004349	-0.011155	-0.006806	Aring
C6	0.331069	0.331583	0.000514	0.316536	0.315235	-0.001301	Aring
C7	0.612100	0.648991	0.036891	0.638474	0.680784	0.04231	Bring
C8	0.064355	-0.026100	-0.090455	0.056662	-0.029508	-0.08617	Bring
C9	0.137864	0.140713	0.002849	0.172881	0.159795	-0.013086	Bring
C10	0.290258	0.251294	-0.038964	0.343024	0.308807	-0.034217	Bring
C11	-0.245186	-0.237302	0.007884	-0.124810	-0.128455	-0.003645	Ering
C12	0.479354	0.479139	-0.000215	0.575832	0.577264	0.001432	Ering
C13	-0.269137	-0.278537	-0.0094	-0.817658	-0.847987	-0.030329	Ering
C14	-0.172986	-0.181452	-0.008466	-0.035328	-0.053491	-0.018163	Ering
C15	-0.176796	-0.166278	0.010518	-0.057812	-0.054904	0.002908	Cring
C16	0.247269	0.254205	0.006936	0.180954	0.204254	0.0233	Cring
C17	-0.709024	-0.678052	0.030972	-0.724300	-0.701763	0.022537	Cring
C18	0.204800	0.231368	0.026568	0.188910	0.207507	0.018597	Cring
C19	0.503472	0.537245	0.033773	0.554952	0.591252	0.0363	Dring
C20	-0.596791	-0.618221	-0.02143	-0.476767	-0.487142	-0.010375	Dring
C21	0.162897	0.138655	-0.024242	-0.033664	-0.049040	-0.015376	Dring
C22	-0.079236	-0.055673	0.023563	0.027591	0.059752	0.032161	Dring
C23	0.182053	0.204976	0.022923	0.190980	0.219629	0.028649	COO(E)
O24	-0.485548	-0.481817	0.003731	-0.416422	-0.418421	-0.001999	
C25	-0.133400	-0.151280	-0.01788	-0.330996	-0.350877	-0.019881	CH2OH(E)
O26	-0.154271	-0.155872	-0.001601	-0.118545	-0.119356	-0.000811	
C27	0.469864	0.471714	0.00185	0.916604	0.945498	0.028894	COOH(E)
O28	0.009218	0.006431	-0.002787	0.098245	0.093744	-0.004501	
O29	-0.194959	-0.175522	0.019437	-0.190087	-0.170048	0.020039	
C30	0.052159	0.066527	0.014368	0.054648	0.072272	0.017624	CH2OH(D)
O31	-0.199542	-0.196238	0.003304	-0.200179	-0.196240	0.003939	
C32	0.372912	0.373851	0.000939	0.143192	0.128645	-0.014547	COO(D)
O33	-0.497176	-0.493291	0.003885	-0.424782	-0.420942	0.00384	
O34	-0.205826	-0.207597	-0.001771	-0.202428	-0.202038	0.00039	
O35	-0.515567	-0.512249	0.003318	-0.463130	-0.461858	0.001272	
O36	-0.446938	-0.450102	-0.003164	-0.380734	-0.385072	-0.004338	
O37	-0.477307	-0.480515	-0.003208	-0.434550	-0.441225	-0.006675	
MET38	1.510761	1.511652	0.000891	1.131888	1.130066	-0.001822	

2) Inter Fragment Charge Transfer IFCT (Sang, S.; Chen, F.; Zhang, C, Int. J. Quantum. Chem 121(6) e26522(2021) https://doi.org/10.1002/qua.26522

A) IFCT for UFQD-Cd2+ without explicit water

Ring A: atoms 1,4,5,6; ring B: 2,3,7,8,9,10; ring C: 15,16,17,18; ring D: 19,20,21,22; ring E: 11,12,13,14; Cd: 38. See figures/tables S1 for atom numbering

Input atom indices for fragment 1, e.g. 1,4,8-12,15
1,4,5,6
Input atom indices for fragment 2, e.g. 1,4,8-12,15
2,3,7,8,9,10
Input atom indices for fragment 3, e.g. 1,4,8-12,15
15,16,17,18
Input atom indices for fragment 4, e.g. 1,4,8-12,15
19,20,21,22
Input atom indices for fragment 5, e.g. 1,4,8-12,15
11,12,13,14
Input atom indices for fragment 6, e.g. 1,4,8-12,15
38
Contribution of each fragment to hole and electron:
1 Hole: 6.37 % Electron: 7.06 %
2 Hole: 23.94 % Electron: 39.76 %
3 Hole: 30.09 % Electron: 18.32 %
4 Hole: 23.60 % Electron: 17.32 %
5 Hole: 10.51 % Electron: 14.11 %
6 Hole: 0.00 % Electron: 0.00 %

Variation of population number of fragment 1: 0.00523
Variation of population number of fragment 2: 0.14455
Variation of population number of fragment 3: -0.11737
Variation of population number of fragment 4: -0.06427
Variation of population number of fragment 5: 0.03186
Variation of population number of fragment 6: 0.00000

Intrafragment electron redistribution of fragment 1: 0.00450
Intrafragment electron redistribution of fragment 2: 0.09518
Intrafragment electron redistribution of fragment 3: 0.05513
Intrafragment electron redistribution of fragment 4: 0.04088
Intrafragment electron redistribution of fragment 5: 0.01484

Intrafragment electron redistribution of fragment 6: 0.00000

Transferred electrons between fragments:

1 -> 2: 0.02531	1 <- 2: 0.01690	Net 1 -> 2: 0.00841
1 -> 3: 0.01167	1 <- 3: 0.02124	Net 1 -> 3: -0.00957
1 -> 4: 0.01103	1 <- 4: 0.01666	Net 1 -> 4: -0.00564
1 -> 5: 0.00899	1 <- 5: 0.00742	Net 1 -> 5: 0.00156
1 -> 6: 0.00000	1 <- 6: 0.00000	Net 1 -> 6: 0.00000
2 -> 3: 0.04387	2 <- 3: 0.11961	Net 2 -> 3: -0.07574
2 -> 4: 0.04146	2 <- 4: 0.09384	Net 2 -> 4: -0.05238
2 -> 5: 0.03379	2 <- 5: 0.04180	Net 2 -> 5: -0.00801
2 -> 6: 0.00000	2 <- 6: 0.00000	Net 2 -> 6: 0.00000
3 -> 4: 0.05211	3 <- 4: 0.04325	Net 3 -> 4: 0.00885
3 -> 5: 0.04246	3 <- 5: 0.01927	Net 3 -> 5: 0.02320
3 -> 6: 0.00000	3 <- 6: 0.00000	Net 3 -> 6: 0.00000
4 -> 5: 0.03332	4 <- 5: 0.01821	Net 4 -> 5: 0.01511
4 -> 6: 0.00000	4 <- 6: 0.00000	Net 4 -> 6: 0.00000
5 -> 6: 0.00000	5 <- 6: 0.00000	Net 5 -> 6: 0.00000

Delta-r index: 1.39682 (see ref. 32). $\Delta r \geq 1.5$ Bohr is the threshold to separate a valence transition from a charge-transfer transition

Lambda index: 0.84525

Transition dipole moment: 3.037 a. u.

B) IFCT for UFQD-Pb2+ without explicit water

Ring A: atoms 1,4,5,6; ring B: 2,3,7,8,9,10; ring C: 15,16,17,18; ring D: 19,20,21,22; ring E: 11,12,13,14; Pb: 38. See figures/tables S2 for numbering

Input atom indices for fragment 1, e.g. 1,4,8-12,15

1,4,5,6

Input atom indices for fragment 2, e.g. 1,4,8-12,15

2,3,7,8,9,10

Input atom indices for fragment 3, e.g. 1,4,8-12,15

15,16,17,18

Input atom indices for fragment 4, e.g. 1,4,8-12,15

19,20,21,22

Input atom indices for fragment 5, e.g. 1,4,8-12,15

11,12,13,14

Input atom indices for fragment 6, e.g. 1,4,8-12,15

38

Contribution of each fragment to hole and electron:

```
 1  Hole:   7.54 %     Electron:   6.54 %
 2  Hole:  24.09 %     Electron:  38.87 %
 3  Hole:  29.30 %     Electron:  17.67 %
 4  Hole:  22.02 %     Electron:  13.39 %
 5  Hole:  11.68 %     Electron:  19.32 %
 6  Hole:   0.00 %     Electron:   0.62 %
```

```
Variation of population number of fragment  1:  -0.01081
Variation of population number of fragment  2:   0.13559
Variation of population number of fragment  3:  -0.11529
Variation of population number of fragment  4:  -0.08553
Variation of population number of fragment  5:   0.07022
Variation of population number of fragment  6:   0.00583
```

```
Intrafragment electron redistribution of fragment  1:   0.00493
Intrafragment electron redistribution of fragment  2:   0.09365
```

Intrafragment electron redistribution of fragment 3: 0.05179
Intrafragment electron redistribution of fragment 4: 0.02949
Intrafragment electron redistribution of fragment 5: 0.02258
Intrafragment electron redistribution of fragment 6: 0.00000

Transferred electrons between fragments:

1 -> 2: 0.02931	1 <- 2: 0.01576	Net 1 -> 2: 0.01356
1 -> 3: 0.01333	1 <- 3: 0.01917	Net 1 -> 3: -0.00584
1 -> 4: 0.01010	1 <- 4: 0.01440	Net 1 -> 4: -0.00430
1 -> 5: 0.01457	1 <- 5: 0.00764	Net 1 -> 5: 0.00693
1 -> 6: 0.00047	1 <- 6: 0.00000	Net 1 -> 6: 0.00046
2 -> 3: 0.04257	2 <- 3: 0.11391	Net 2 -> 3: -0.07133
2 -> 4: 0.03227	2 <- 4: 0.08558	Net 2 -> 4: -0.05332
2 -> 5: 0.04655	2 <- 5: 0.04542	Net 2 -> 5: 0.00113
2 -> 6: 0.00149	2 <- 6: 0.00001	Net 2 -> 6: 0.00148
3 -> 4: 0.03925	3 <- 4: 0.03891	Net 3 -> 4: 0.00034
3 -> 5: 0.05662	3 <- 5: 0.02065	Net 3 -> 5: 0.03597
3 -> 6: 0.00181	3 <- 6: 0.00001	Net 3 -> 6: 0.00181
4 -> 5: 0.04254	4 <- 5: 0.01565	Net 4 -> 5: 0.02689
4 -> 6: 0.00136	4 <- 6: 0.00000	Net 4 -> 6: 0.00136
5 -> 6: 0.00072	5 <- 6: 0.00001	Net 5 -> 6: 0.00072

Delta-r index: 1.46032 Bohr (see ref. 32). $\Delta r \geq 1.5$ Bohr is the threshold to separate a valence transition from a charge-transfer transition

Lambda index: 0.83280

Transition dipole moment: 2.917 a. u.
